# Chromosomal Locations of 5S and 45S rDNA in *Gossypium* Genus and Its Phylogenetic Implications Revealed by FISH

**DOI:** 10.1371/journal.pone.0068207

**Published:** 2013-06-27

**Authors:** Yimei Gan, Fang Liu, Dan Chen, Qiong Wu, Qin Qin, Chunying Wang, Shaohui Li, Xiangdi Zhang, Yuhong Wang, Kunbo Wang

**Affiliations:** 1 State Key Laboratory of Cotton Biology (China)/Institute of Cotton Research of Chinese Academy of Agricultural Sciences, Anyang, Henan, P. R. China; 2 Institute of Tropical Bioscience and Biotechnology of Chinese Academy of Tropical Agricultural Sciences/Key Laboratory of Biology and Genetic Resources of Tropical Crops, Ministry of Agriculture (China), Haikou, Hainan, P. R. China; 3 Haikou Experimental Station of Chinese Academy of Tropical Agricultural Sciences, Haikou, Hainan, P. R. China; East Carolina University, United States of America

## Abstract

We investigated the locations of 5S and 45S rDNA in *Gossypium* diploid A, B, D, E, F, G genomes and tetraploid genome (AD) using multi-probe fluorescent in situ hybridization (FISH) for evolution analysis in *Gossypium* genus. The rDNA numbers and sizes, and synteny relationships between 5S and 45S were revealed using 5S and 45S as double-probe for all species, and the rDNA-bearing chromosomes were identified for A, D and AD genomes with one more probe that is single-chromosome-specific BAC clone from *G. hirsutum* (A_1_D_1_). Two to four 45S and one 5S loci were found in diploid-species except two 5S loci in *G* *. incanum* (E_4_), the same as that in tetraploid species. The 45S on the 7th and 9th chromosomes and the 5S on the 9th chromosomes seemed to be conserved in A, D and AD genomes. In the species of B, E, F and G genomes, the rDNA numbers, sizes, and synteny relationships were first reported in this paper. The rDNA pattern agrees with previously reported phylogenetic history with some disagreements. Combined with the whole-genome sequencing data from *G* *. raimondii* (D_5_) and the conserved cotton karyotype, it is suggested that the expansion, decrease and transposition of rDNA other than chromosome rearrangements might occur during the *Gossypium* evolution.

## Introduction

Cotton ( *Gossypium* ) is an important economic fiber crop. The genus of *Gossypium* comprises about 50 species throughout tropical and subtropical regions of the world, including five tetraploid (2n=4x=52) species and about 45 diploid (2n=2x=26) species. The taxonomic and evolution study on *Gossypium* genus has been an important subject of investigation due to its economic significance. In the late 1800’s or early 1900’s, the taxonomy was mainly based on morphological characteristics and geographical distributions, however, it has been confusion due to un-consensus characteristics used by different taxonomists. With development of cytological methods, the diploid species have been classified genetically into seven genome types, i.e. A, B, C, D, E, F, G and K genomes [1,2]. The evolutionary history of *Gossypium* genus was reconstruct based on geography, morphology, cytogenetics and molecular data. However, due to the continuous recombination and exchange, great differences existed between existing cotton species and their ancestors in terms of physiological feature, agronomic trait and morphology. Therefore, more interpretation about the phylogenetic and interspecific evolution in *Gossypium* genus is quite necessarily to be clarified.

Ribosomal DNA (rDNA) has highly conserved repetitive sequences in the plant genome, and the polymorphism or conservatism of their copy number and chromosomal localization are visual and comparative [3–5]. By comparing the number and distribution characteristics of rDNA sites on the chromosomes among species, interspecific phylogenetic relationships and the related mechanism of speciation and chromosomal evolution could be revealed [6]. Recently, the physical FISH location of rDNA in plants have provided much information to the evolutional relationship of many close species and the origin of allopolyploid [7–12].

In genus of *Gossypium* , research on rDNA location in the early days was mainly focused on *G. hirsutum* (upland cotton) due to its economic importance and to the abundant genetic materials created. 5S rDNA and 18S-28S rDNA were located to chromosomes of *G. hirsutum* by FISH on chromosomes of the meiosis metaphase [13–15]. Later, the number and copy number of 5S and 18S-28S rDNA in tetraploid *G. hirsutum*, diploid species of A and D genomes, were revealed by FISH on the metaphase chromosomes of mitosis [16]. Recently, the number and copy numer of 5S and 45S rDNA, the 5S-bearing and 45S-bearing chromosomes of other tetraploid species and diploid species of A and D genomes have been revealed [17–19].

In order to further understand the cytogenetics and evolution of *Gossypium* genus, the distribution of 5S and 45S rDNA was analyzed by cocktail FISH for the four tetraploid species, as well as 13 diploid species and one variation representing diploid A, B, D, E, F and G genome. Combined with rDNA distribution in previous reports [17–19], the chromosome evolution of rDNA loci of *Gossypium* genus would be determined. Also, the phylogenetic implication based on rDNA patterns could be inferred to gain further insight into the evolutionary history of *Gossypium* genomes.

## Materials and Methods

### Plant materials and clones

The species and their genomes and accessions (cultivars) used in this study were shown in Table 1. The plant materials are maintained perennially in the National Wild Cotton Nursery in Sanya City, Hainan Island, sponsored and owned by the Institute of Cotton Research of Chinese Academy of Agricultural Sciences (ICR-CAAS), and at the same time, some of them are as well conserved in pots in greenhouse of ICR-CAAS at Anyang City, Henan Province, China.

**Table 1:** *Gossypium* species and their accessions used.

**Species/variant**	**Genome**	**Accession/cultivar**	**Accession No. in nursery**	**Pot No. in greenhouse**
*G. hirsutum*	A_1_D_1_	TM-1		
*G. barbadense*	A_2_D_2_	Pima 90-53		
* G. tomentosum *	A_3_D_3_		H0701306	H0701301
* G. mustelinum *	A_4_D_4_	A_4_D_4_-9	P0811807	H0804201
* G. laxum *	D_9_		P0601001	
* G. schwendimanii *	D_11_		P0602110	
* G. gossypioides *	D_6_	D_6_-2	P0814608	H0006401
* G. raimondii *	D_5_	D_5_-2	P0811506	H0006301
* G. herbaceum *	A_1_	Hongxingcaomian		
* G. herbaceum var. africanum*	A_1-a_		D2030202	H0000101
* G. anomalum *	B_1_		P0601305	H0000201
* G. capitis -viridis*	B_3_	B_3_-1		H0004601
* G. somalense *	E_2_	E_2_-3	P0815401	H0007001
* G. areysianum *	E_3_		P0601809	H0001901
* G. incanum *	E_4_	E_4_-4	P0815512	
* G. longicalyx *	F_1_	F_1_-3	P0815709	H0007201
* G. bickii *	G_1_	G_1_-1	P0815801	
* G. nelsonii *	G_3_	G_3_-1	P0816209	H0807601

For diploid D genome species and D_t_ subgenome of tetraploid species, four types of probes were used, including 5S rDNA, 45S rDNA, BAC clone 150D24 and some D_h_ chromosome-specific BAC clones. For diploid A genome and A_t_ subgenome of tetraploid species, three types of probes were used, including 5S rDNA, 45S rDNA and some A_h_ chromosome-specific BAC clones. For the diploid B, E, F and G genome species, only 5S rDNA and 45S rDNA were used. The 5S and 45S rDNA derived from *Arabidopsis thaliana* were kindly provided by Professor Yunchun Song of Wuhan University, China. The BAC clone 150D24 which contains centromere-specific repeats in D subgenome and D genome of *Gossypium* was screened from Pima 90-53 BAC library [20] to indicate centromere position. The A_h_ (D_h_) chromosome-specific BAC clones used to identify individual chromosome were kindly provided by Professor Tianzhen Zhang of Nanjing Agricultural University, China [21].

### DNA probes preparation

The probes 5S, 45S rDNA and BAC DNA were isolated using a standard alkaline extraction [22]. 45S rDNA and BAC clone 150D24 were labeled by standard Dig-nick translation reactions, whereas 5S rDNA and some A_h_ (D_h_) subgenome chromosome-specific BAC clones [21] were labeled with Biotin-nick translation reactions, according to the manufacturer’s instructions (Roche Diagnostics, USA).

### Chromosome preparation and FISH

Preparation of mitotic chromosomes and the FISH procedure were conducted according to [23] with some modifications. Digoxigenin-labeled and biotin-labeled probes were detected by anti-digoxigenin-rhodamine (red) and avidin-fluorescein (green) (Roche Diagnostics, USA), respectively. For conducting the probe-cocktail mixture, gDNA was used as block DNA instead of Cot-1 DNA. The dose of block DNA was 200 times of the chromosome-specific BAC DNA. Chromosomes were counterstained by 4’, 6-diamidino-2- phenylindole (DAPI) in the antifade VECTASHIELD solutions (Vector Laboratories, Burlingame, CA). The hybridization signals were observed using a fluorescence microscope (Leica MRA2) with a charge-coupled device (CCD) camera (Zeiss) and arranged using Adobe Photoshop 7.0.

## Results

### The number of 5S and 45S rDNA in *Gossypium* genus

**Figure 1:**
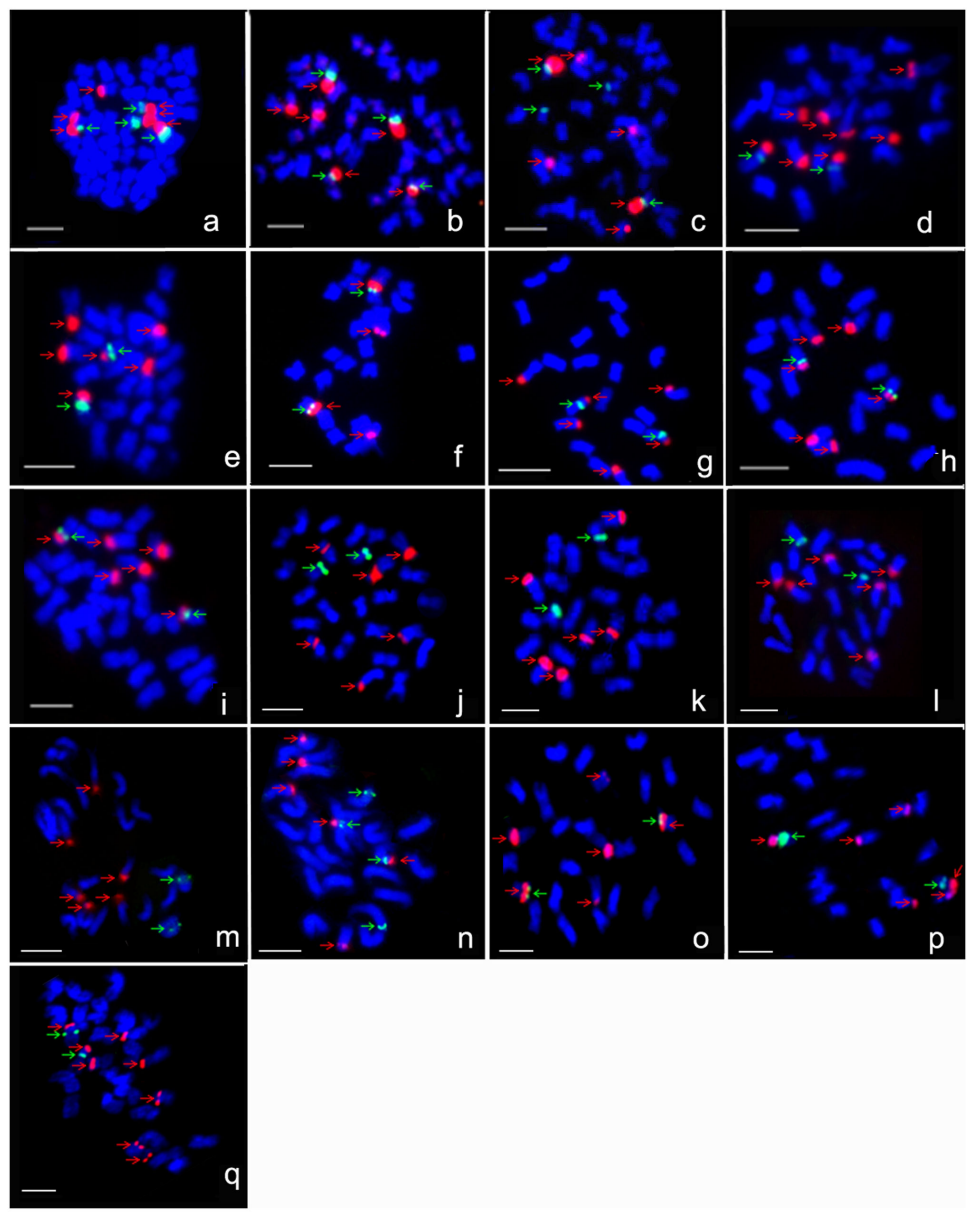
The number of rDNA in AD, D, A, B, E, F and G genomes. 5S rDNA: green fluorescence signals marked with green arrows; 45S rDNA: red fluorescence signals marked with red arrows. a: A_1_D_1_, b: A _3_D_3_, c: A _4_D_4_, d: D_5_, e: D_6_, f: D_9_, g: D_11_, h: A_1_, i: A_1-a_, j: B_1_, k: B_3_, l: E_2_, m: E_3_, n: E_4_, o: F_1_, p: G_3_, q: G_1_. Bar =5 µm.

Three 45S rDNA loci and two 5S rDNA loci were detected in all three tetraploid species (Figure 1 a, b, c). Similarly, the number of 45S rDNA loci was detected three, two, four and three in D_5_, D_6_, D_9_, D_11_, respectively, while only one 5S rDNA locus was observed in the four D genome species (Figure 1d–1g). In A_1_ and its variant A_1-a_, three 45S loci and one 5S rDNA locus were found, respectively (Figure 1 h, 1i). In B_1_, B_3_, E_2_, E_3_, F_1_ and G_3_, three 45S loci and one 5S rDNA locus were observed (Figure 1j–1m, 1p), while three 45S loci and two 5S rDNA loci in E_4_ (Figure 1n) as well as four 45S loci and one 5S rDNA locus in G_1_ were observed (Figure 1q).

### The location of 5S and 45S rDNA in *Gossypium* genus

**Figure 2:**
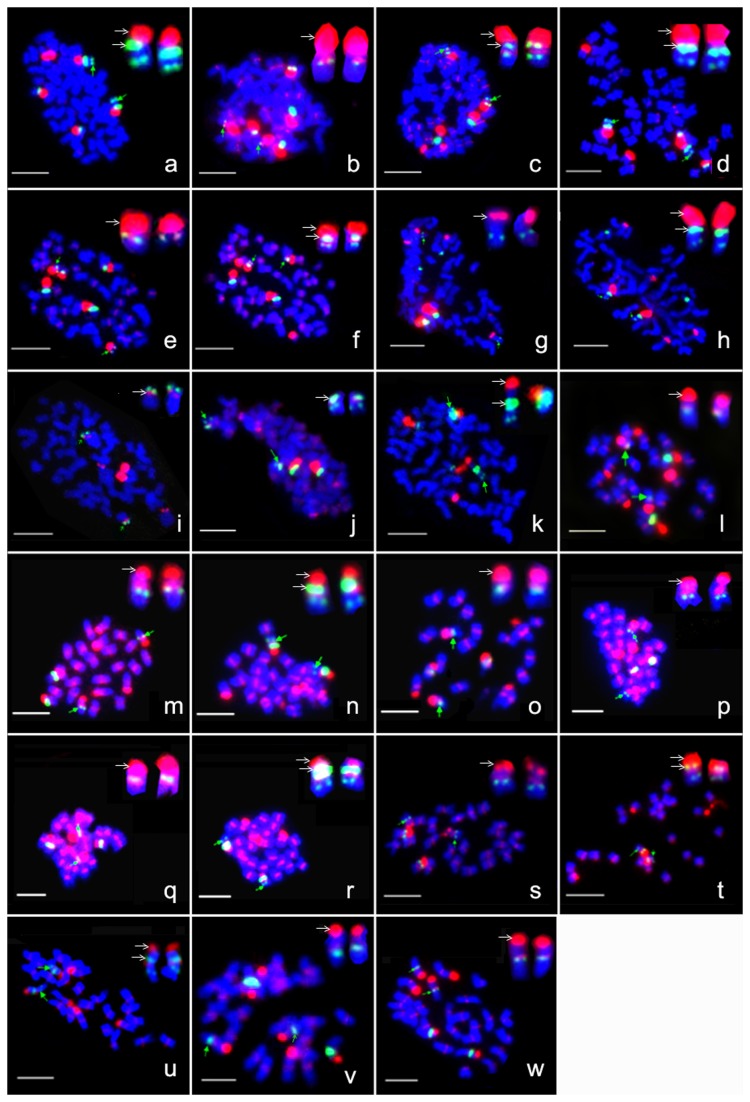
Locations of 5S and 45S rDNA in four tetraploid and four D-genome species. green and weak fluorescence signals with green arrow; 5S rDNA: green fluorescence signals; 45S rDNA: red fluorescence signals. For D-genome species and D-subgenome, the short arm and the long arm were distinguished by the location of 150D24 with red fluorescence signals on intercalary chromosomes. Marked chromosomes with green arrow were enlarged at the top-right corner with the short arm on the top, and the 45S or 5S signals were marked with white arrow. Bar =5µm. a–c: FISH images with 45S on chromosomes A _h_09, D _h_07 and D _h_09 and that with 5S on chromosomes A _h_09 and D _h_09 for A _1_D_1_, respectively. d–f: FISH images with 45S on chromosomes A _tt_09, D _tt_07 and D _tt_09 and that with 5S on chromosomes A _tt_09 and D _tt_09 for A _3_D_3_, respectively. g–j: FISH images with 45S on chromosomes A _m_07, A _m_09 and A _m_08 (g, h, i) and that with 5S on chromosomes A _m_09 and D _m_09 (h, j) for A _4_D_4_, respectively. k: FISH images with 45S and 5S on chromosome A _b_09 of A _2_D_2_. l–o: FISH images with 45S and 5S on chromosomes D_9_05, D_9_07, D_9_09 and D_9_12 and that with 5S on chromosome D _9_09 for D_9_, respectively. p–r: FISH images with 45S and 5S on chromosomes D_11_05, D_11_07 and D_11_09 and that with 5S on the chromosome D _11_09 for D_11_, respectively. s, t: FISH images with 45S and 5S on chromosomes D_6_07 and D_6_09 and that with 5S on chromosome D _6_09 for D_6_, respectively. u–w: FISH images with 45S and 5S on chromosomes D_5_09, D_5_02 and D_5_11 and that with 5S on chromosome D _5_09 for D_5_, respectively.

To further identify the rDNA locations specific to individual chromosomes or even to arms in D subgenome of tetraploid and D genome cottons, the individual chromosome-specific BAC clones and a D genome centromere-specific BAC clone (150D24) as BAC-FISH probes were used in the experiments. And for D_t_ subgenome of tetraploid, the individual chromosome BAC clones was used as BAC-FISH probes. Figure 2a–2w showed the 45S and 5S rDNA locations specific to individual chromosomes or even to arms in the three tetraploid cottons (A_1_D_1_, A_3_D_3_, A_4_D_4_ and A_2_D_2_) and the four D genome cottons (D_9_, D_11_, D_6_, D_5_). In both A_1_D_1_ (Figure 2a–2c) and A_3_D_3_ (Figure 2d–2f), 45S and 5S rDNA, syntenic with BAC clones specific to chromosomes A_h_09, D_h_07 and D_h_09 (h indicates A_1_D_1_), respectively, were located to the corresponding chromosomes and chromosomal arms. According to the homology within D subgenomes, chromosomes bearing with 45S locus of A_3_D_3_ were named as A_tt_09, D_tt_07 and D_tt_09 (tt indicates A_3_D_3_), respectively. So that in these two species, three 45S loci were observed at the end of the short arm of chromosomes, whereas two 5S loci were co-localized with the 45S locus and was found interstitial on the short arm of chromosomes A_t_09 and D_t_09 (t indicates tetraploid species), respectively, suggesting a synteny relationship for 5S and 45S rDNA. In A_4_D_4_, three 45S loci, syntenic with BAC clones specific to chromosomes A_h_07, A_h_09 and A_h_08 respectively were found located at the end of the short arm of chromosomes of A subgenome (Figure 2g–2i), while the two 5S loci, syntenic with BAC clones specific to chromosomes A_h_09 and D_h_09, respectively, were located to the end of the short arm of chromosomes (Figure 2h, 2j). Likewise, chromosomes bearing 45S and 5S rDNA loci were named as A_m_07, A_m_09, A_m_08 and D_m_09, respectively (m indicates A_4_D_4_). And so, 5S and 45S rDNA, being positioned on the chromosome A _m_09, showed a synteny relationship, and the other 5S rDNA was positioned on the on the chromosome D _m_09 showing no synteny relationship with any of 45S loci. In addition, 5S and 45S rDNA on the A subgenome of A_2_D_2_ was located to the same chromosome A _b_09 (b indicates A_2_D_2_) (Figure 2k).

Figure 2l–2w showed the 45S and 5S rDNA locations specific to individual chromosomes or even to arms in the four D genome cottons (D_9_, D_11_, D_6_, D_5_). In D_9_, 45S and 5S rDNA, syntenic with BAC clones specific to chromosomes D_h_05, D_h_07, D_h_09 and D_h_12, respectively, were located to the corresponding chromosomes and chromosomal arms (Figure 2l–2o). According to the homology between D genomes and D subgenome of A_1_D_1_, chromosomes bearing 45S locus of D_9_ were named as D_9_05, D_9_07, D_9_09 and D_9_12, respectively. The result showed that four 45S rDNA loci were found at the end of short arm of chromosomes D_9_05, D_9_07, D_9_09 and D_9_12, with one 5S rDNA locus was found interstitial to the short arm of chromosome D _9_09. Likewise, 45S and 5S rDNA, syntenic with BAC clones specific to chromosomes D_h_05, D_h_07 and D_h_09, were found respectively located to the corresponding chromosomes and chromosomal arms of D_11_ (Figure 2p–2r). Chromosomes bearing with 45S locus of D_11_ were named as D_11_05, D_11_07 and D_11_09, respectively. Three 45S rDNA loci were showed at the end of short arm of chromosomes D_11_05, D_11_07 and D_11_09, and one 5S rDNA locus was found interstitial to the short arm of chromosome D _11_09. Also, 45S and 5S rDNA, syntenic with BAC clones specific to chromosomes D_h_07 and D_h_09, respectively, were located to the corresponding chromosomes and chromosomal arms of D_6_ (Figure 2s, 2t). Chromosomes bearing with 45S locus of D_6_ were named as D_6_07 and D_6_09, respectively. Therefore, two 45S rDNA loci were seen at the end of short arm of chromosomes D_6_07 and D_6_09, and one 5S rDNA locus was found interstitial to the short arm of chromosome D _6_09. In D_5_, two 45S loci and one 5S rDNA, syntenic with BAC clones specific to chromosomes D_h_09 and D_h_11, respectively, were located to the corresponding chromosomes (Figure 2u, 2w). Since the chromosome bearing the third 45S could not be identified with BAC clones derived from *G. hirsutum* (Gan unpublished), the third 45S was therefore identified to chromosome 02 (Figure 2v) with BAC clone screened from A_2_D_2_ (Qinqin unpublished). According to the homology between D genome and D subgenome, chromosomes bearing with 45S locus of D_5_ were named as D_5_09, D_5_11 and D_5_02, respectively. And, three 45S loci were shown at the end of short arm of chromosomes D_5_09, D_5_11 and D_5_02, while one 5S rDNA locus was found interstitial to the short arm of chromosome D _5_09.

**Figure 3:**
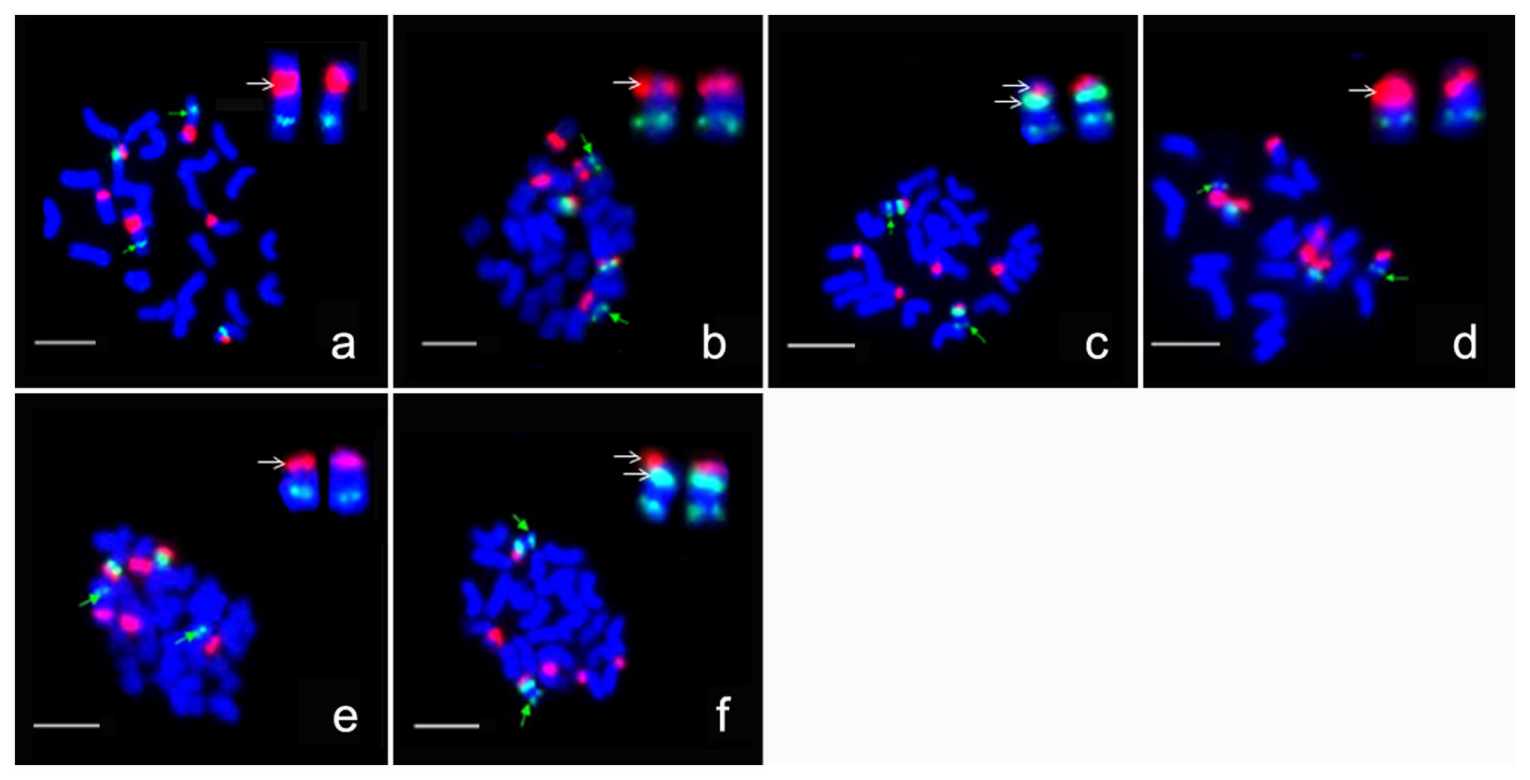
Locations of 5S and 45S rDNA in two A-genome species by dual-FISH. BAC DNA: green and weak fluorescence signals with green arrow; 5S rDNA: green fluorescence signals; 45S rDNA: red fluorescence signals. Marked chromosomes with green arrow were enlarged at the top-right corner with the short arm on the top, and the 45S or 5S signal were marked with white arrow. Bar =5µm. a–c: FISH images with 45S and 5S on chromosomes A _1_05, A _1_07 and A _1_09 and that with 5S on chromosome A _1_09 for A_1_, respectively. d–f: FISH images with 45S and 5S on chromosomes A _1-a_05, A _1-a_07 and A _1-a_09 and that with 5S on chromosome A _1-a_09 for A_1-a_, respectively.

Figure 3a–3f showed the 45S and 5S rDNA locations specific to individual chromosomes in A_1_ and A_1-a_. Three 45S and one 5S rDNA, syntenic with BAC clones specific to chromosomes A_h_05, A_h_07 and A_h_09, were located to the corresponding chromosomes. According to the homology between A genomes and A subgenome, chromosomes bearing 45S locus were named as A_1_05, A_1_07 and A_1_09 (for A_1_) (Figure 3a–3c), A_1-a_05, A_1-a_07 and A_1-a_09 (for A_1-a_) (Figure 3d–3f), respectively. Therefore, in both A_1_ and A_1-a_, three 45S rDNA loci were revealed at the end of short arm of chromosomes A_1_05 (A_1-a_05), A_1_07 (A_1-a_07) and A_1_09 (A_1-a_09), and one 5S locus was located on chromosomes A_1_09 (Figure 3c) and A_1-a_09 (Figure 3c), respectively.

There is limited genomic information available for species of B, E, F and G genomes, and the chromosome identification for BAC clones is unavailable either, and so the location of 5S and 45S rDNA has had been identified by using rDNA-FISH with only 5S and 45S rDNA probed. In B_1_ and B_3_, one 5S locus and two 45S loci were observed at the end of chromosomes, while one 45S locus was found nearby centromere, displaying the satellite-intermediate type, which is extremely rare in *Gossypium* species (Figure 1j, 1k). In E_2_, E_3_, E_4_, 5S and 45S were both located at the end of chromosomes (Figure 1l–1n). In F_1_, two 45S rDNA loci and the third 45S rDNA locus syntenic with 5S rDNA were located to the end and near centromere of chromosomes, respectively, while the 5S rDNA locus of F_1_ was positioned outside the 45S rDNA and to the end of chromosome (Figure 1o). In G_1_ and G_3_, 45S and 5S rDNA were located at the end and near centromere of chromosomes, respectively (Figure 1p, 1q). And the relationship between 5S and 45S is nonsyntenic in B_1_, B_3_, E_2_ and E_3_, while it showed syntenic in F_1_, G_1_ and G_3_. Among two 5S loci in E_4_, one is synteny with 45S rDNA while the other one is not synteny with 45S rDNA.

### Chromosome distribution of rDNA in *Gossypium* genus

**Figure 4:**
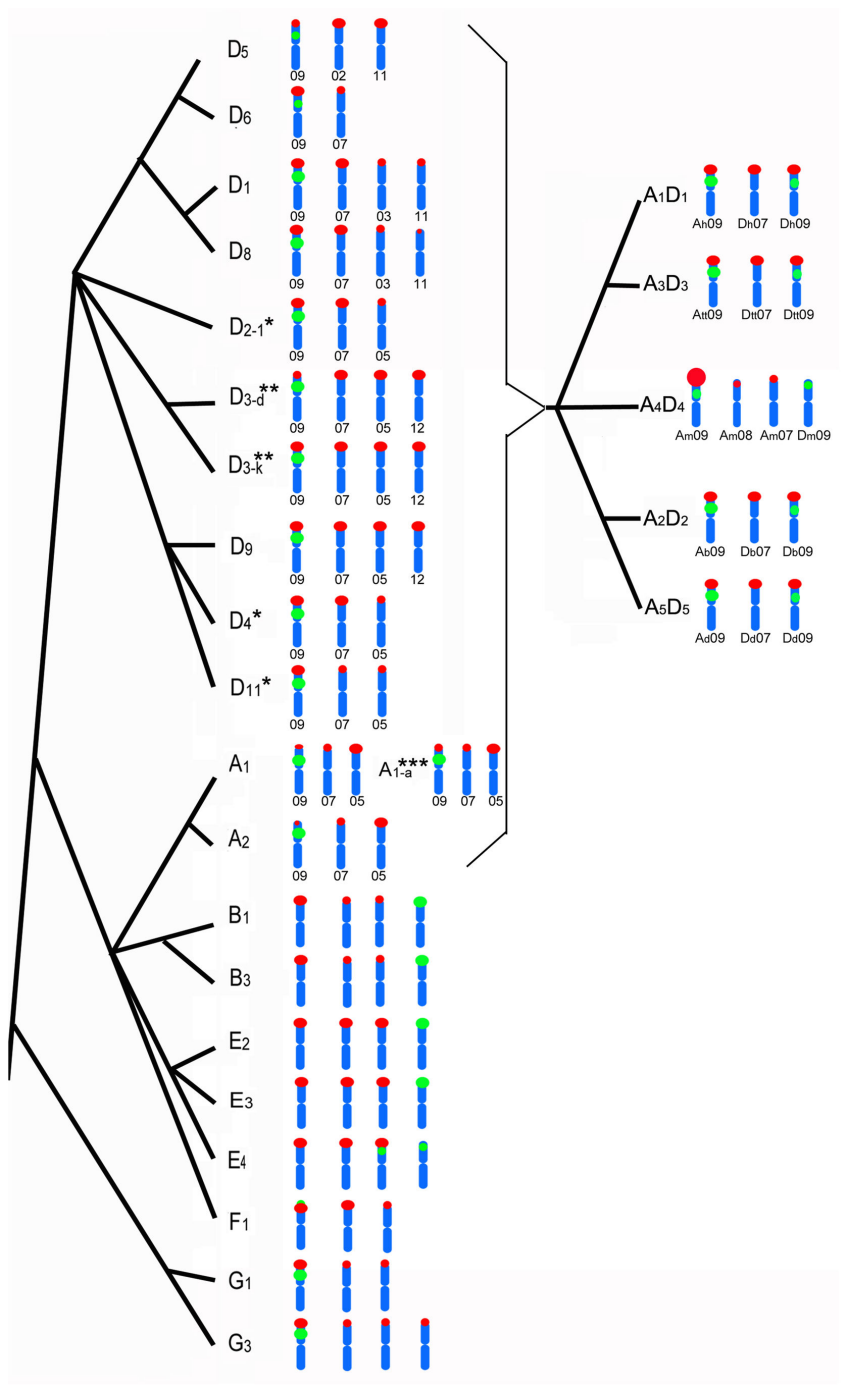
Simplified phylogeny of *Gossypium* genus included in idiograms chromosomes bearing 5S and 45S rDNA signals. The phylogeny is according to the reference of Wendel [24]. The words under the chromosomes indicated the chromosomes bearing the rDNA locus. 5S rDNA: green signals; 45S rDNA: red signals. * and ** indicate the differences according 45S rDNA pattern in the present study from previous report [24], respectively; *** indicates *G. herbaceum* *var.* *africanum* .

In order to compare and analyze the evolutional relationship among cotton species, the chromosomal distribution of 5S and 45S for 17 species and one variant in the present study and 8 species presented in previous papers [17–19] are summarized in Table 2 and displayed schematically in Figure 4.

**Table 2:** Distribution of rDNA in *Gossypium* genus.

**Genome**	**Species(short name)**	**(No.) Chr. bearing 45S**	**(No.) Chr. bearing 5S**	**Relationship between 5S and 45S**	**Source**
AD	*G. hirsutum* (A_1_D_1_)	(3)A_h_09, D_h_07, D_h_09	(2)A_h_09, D_h_09	synteny	This study
	*G* *. barbadense* (A_2_D_2_)	(3)A_b_09, D_b_07, D_b_09	(2)A_b_09, D_b_09	synteny	[17]
	*G* *. tomentosum* (A_3_D_3_)	(3)A_tt_09, D_tt_07, D_tt_09	(2)A_to_09,D_to_09	synteny	This study
	*G* *. mustelinum* (A_4_D_4_)	(3)A_m_09, A_m_07, A_m_08	(2)A_m_09,D_m_09	synteny/nonsynteny	This study
	*G* *. darwinii* (A_5_D_5_)	(3)A_d_09, D_d_07, D_d_09	(2)A_d_09, D_d_09	synteny	[18]
A	*G* *. herbaceum* (A_1_)	(3) 09, 07, 05	(1) 09	synteny	This study
	*G. herbaceum* *var. Africanum* (A_1-a_)	(3) 09, 07, 05	(1) 09	synteny	This study
	*G* *. arboreum* (A_2_)	(3) 09, 07, 05	(1)09	synteny	[19]
D	*G* *. thurberi* (D_1_)	(4) 09, 07, 03, 11	(1) 09	synteny	[17]
	*G* *. armourianum* (D_2-1_)	(3) 09, 07, 05	(1) 09	synteny	[18]
	*G* *. davidsonii* (D_3-d_)	(4) 09, 07, 05, 12	(1) 09	synteny	[18]
	*G* *. klotzschianum* (D_3-k_)	(4) 09, 07, 05, 12	(1) 09	synteny	[18]
	*G* *. aridum* (D_4_)	(3) 09, 07, 05	(1) 09	synteny	[18]
	*G* *. raimondii* (D_5_)	(3) 09, 11, 02	(1) 09	synteny	This study
	*G* *. gossypioides* (D_6_)	(2) 09, 07	(1) 09	synteny	This study
	*G* *. trilobum* (D_8_)	(4) 09, 07, 03, 11	(1) 09	synteny	[17]
	*G* *. laxum* (D_9_)	(4) 09, 07, 05, 12	(1) 09	synteny	This study
	*G* *. schwendimanii* (D_11_)	(3) 09, 07, 05	(1) 09	synteny	This study
B	*G* *. anomalum* (B_1_)	(3) Unknown	(1) Unknown	nonsynteny	This study
	*G* *. capitis* *-viridis* (B_3_)	(3) Unknown	(1) Unknown	nonsynteny	This study
E	*G* *. somalense* (E_2_)	(3) Unknown	(1) Unknown	nonsynteny	This study
	*G* *. areysianum* (E_3_)	(3) Unknown	(1) Unknown	nonsynteny	This study
	*G* *. incanum* (E_4_)	(3) Unknown	(2) Unknown	synteny/nonsynteny	This study
F	*G* *. longicalyx* (F_1_)	(3) Unknown	(1) Unknown	synteny	This study
G	*G* *. nelsonii* (G_3_)	(3) Unknown	(1) Unknown	synteny	This study
	*G* *. bickii* (G_1_)	(4) Unknown	(1) Unknown	synteny	This study

The 5S rDNA loci maintained high homogeneity in the number (except E_4_), distribution and the copy number among 25 species and one variant, only varied partly in copy numbers in five tetraploid species (Table 2 and Figure 4). In all diploid species except E_4_, only one 5S rDNA locus was found. In tetraploid species and diploid species of A and D genome, all 5S rDNA loci were located to chromosomes 09 (A_t_09/D _t_09/A _g_09/D _g_09). The number of 45S rDNA is conserved in some species but is various in other species, no matter related with their genome resource. Even the same number of 45S loci was observed, they were still positioned to different chromosomes. Beside the number and chromosomes bearing 45S, the copy number (identified by signal intensity) of 45S rDNA was observed to be similar to some extend, but varied to a great extent. They varied in either different species or different chromosomes of the same species. In addition, the syntenic relationship between 5S and 45S rDNA were divided into three groups. One is syntenic for A, D, F and G genome, and the second is nonsyntenic for B and two species of E genome. Then the third one is both syntenic and nonsyntenic in E_4_ which has two 5S rDNA loci.

## Discussion

Genomic and evolutionary researches in *Gossypium* have obtained great progress with morphological characteristics, geographical distribution, cytogenetic and molecular data. And the evolutionary history has been built into phylogenetic tree [24]. However, our understanding of evolution and chromosome structure is still extremely limited. Most of *Gossypium* species have rather small chromosomes, which in many cases are similar in shape and size, and therefore are difficult to be distinguished. In this study, we used chromosome-specific BAC clones from *G. hirsutum*, 5S and 45S rDNA as multiple probes to have FISH located precisely the 5S and 45S rDNA of tetraploid species and species of their donor genome (A and D genome). And species of other genomes are FISH located with 5S and 45S rDNA as double probes. In general, 5S and 45S rDNA were revealed for both conservation and polymorphism in the number of rDNA loci, the number of rDNA repeats, chromosome-bearing rDNA and the synteny relationship between 5S and 45S rDNA. The rDNA pattern is generally in accordance with phylogeny with some disagreements. It is noted that, the chromosomes bearing 5S and 45S rDNA have been identified accurately for A and D genome as well as the tetraploid species using chromosome-specific BAC clones. Therefore, location of 5S and 45S rDNA in our study provided more detailed and comprehensive information for the evolution of *Gossypium* genus than the previous reports, which provided insights into molecular evolutionary changes.

### Chromosomal patterns of 5S and 45S rDNA

The 5S rDNA loci among 25 cotton species (Table 2 & Figure 4) were observed to be highly conserved in this study. Only one 5S rDNA locus was found in all diploid species except E_4_ and two loci in tetraploid species. The high conservation of 5S rDNA is a common phenomenon in plant genera such as *Oryza* genus [11], *Arichis* genus [8], and Quadrifaria group of *Paspalum* genus [25]. The conserved distribution of 5S rDNA might be associated with their location at pericentromeric regions [26], which rarely related to the chromosomal structure rearrangement [27]. Also, the recombination intercalary site of chromosome is much lower than that of ones at the end of chromosome in upland cotton [21].

The distribution of 45S rDNA among 25 cotton species (Table 2 & Figure 4) is both conserved and polymorphic, in accordance with other reports showing similar pattern [11,16]. The number variations of rDNA among plants of the same ploidy level have been attributed to chromosomal rearrangements, transposable events and gene silence [28]. According to the data of the whole genome sequencing of *G* *. raimondii* which is the smallest *Gossypium* species, a high proportion of transposable elements such as the *gypsy* and *copia*-like LTRs were found [29], suggesting that the most possible mechanisms associated with 45S rDNA variation could be transposon mobility. Taking conservative karyotypes of *Gossypium* interspecies into account, major chromosomal structural rearrangements are not frequent among species. Therefore, in *Gossypium* genus, the key mechanism facilitating diversification of 45S rDNA distribution patterns should be considered as transpositions rather than chromosome rearrangements. Moreover, the copy numbers of 45S rDNA loci was discrepant in the different chromosomes of the same species or the corresponding chromosomes of different species. The copy numbers of rDNA repeats might be amplified or decreased by unequal crossing over to the extent that these new sites can be detected by FISH.

Besides the numbers and copy numbers of rDNA, some tendencies of syntenic relationship between 5S and 45S rDNA have been as well indicated. It is syntenic for A, D, F and G genome, but nonsyntenic for B and E genome. The diploid species were divided into Australian species (C, G and K genome), American species (D genome) and Africa-Asian species (A, B, E and F genome), and the last one is considered as original species [30]. Species of B and E genome could be the most original according to the chromosomal pairing analysis in interspecies hybridization [31], electrophoretic analysis of seed protein [32] and phenotypic relationship analysis [33]. According to the evolution pattern, more advanced species evolved from the distribution center to the edge of the expansion. It is considered that Africa species (A, B, E and F genome) originated from Africa which was a distribution centre of cotton [30]. Therefore, the nonsynteny relationship in species of B and E genomes might be related the original types.

### Phylogenetic implications with rDNA pattern for *Gossypium* genus

#### Phylogenetic implications for tetraploid species

The variations in rDNA distribution are with phylogenetic implications, for the closeness of taxa is correlated to the similarity of their rDNA FISH patterns [6]. Theoretically, the rDNA loci of tetraploid cottons should be the sum of rDNA loci of its putative diploid ancestors (A and D genome). In the present study, the sum of 5S rDNA loci of species of A and D genome was equal to that of tetraploid cottons, while 45S rDNA loci decreased in tetraploid cottons. The nonadditive contribution of rDNA during the evolution of polyploidy species has been described in several plant genera [10,16,28,34]. Several hypotheses may explain the reason. Decreases in site number could arise to stabilize new genomes by the formation of translocations with breakpoints proximal to the rDNA sites during the formation of polyploidy [35]. And, deletions may have eliminated loci in modern tetraploid cottons. Also, new 45S rDNA loci may have been formed by transposition of sequences containing rDNA repeats in the modern A and D-diploids.

Additionally, the copy number of 45S rDNA locus on chromosome A _t_09 in tetraploid species is much higher than that of chromosome A _g_09 in donor genome, but that of 5S rDNA locus of D_t_09 in tetraploid species reduced obviously relative to that of D_g_09 in donor genome. It is possible that the copy number of 45S rDNA locus on chromosome A _g_09 in original parental species is very high, but unfortunately the species died out [35]. Or the copy number of 45S rDNA locus on chromosome A _t_09 could increase after the formation of polyploidy or the subsequent evolution. Compared to parental species, 5S rDNA locus in polyploidy species according to the researches on other genus reported tended to eliminate [6,16,34–36]. Some hypotheses may explain why 5S rDNA locus disappeared or their copy number reduced following polyploidization of tetraploid cottons. Firstly, the copy number of 5S rDNA locus could be very low in the original parental species which may extinct [37]. Secondly, the copy number decreased during the course of the formation of tetraploid species. Thirdly, the copy number did not decrease in the modern tetraploid species evolved from the original ones. And the related mechanisms account for the changes could be unequal crossing over, gene exchange and transposons events, and so on [3].

#### Phylogenetic implication for American diploid species (D genome)

Thirteen species of D genome containing six subsections, have received considerable phylogenetic attentions [30,38–40], but evolutionary relationships among these subsections still have not enough evidence [24]. Some evolutionary evidences could be obtained from the rDNA patterns which were revealed both conserved and changeable in ten species of six subsections studied here. The evidence is that it is conserved in the chromosomal location, copy number and synteny relationship of 5S and 45S rDNA on chromosomes D_g_07 and D_g_09 in ten species except D_5_ and D_3-d_ (Table 2 and Figure 4). D_5_, divided into subsection *Austroamericana* Fryxell at the end of D genome [1], the 45S loci on chromosomes D_5_09, D_5_11 and D_5_02 differ greatly with other species (Table 2 and Figure 4), suggesting the greater evolutional history than that of other D-genome species. D_6_ is the sole representative of subsection *Selera* Fryxell with distinctive morphological characteristics [41]. It has only two 45S loci on chromosomes D_6_07 and D_6_09 (Table 2 and Figure 4), which is in agreement with that of D_6_ and could be used as the base of D genome species [34]. D_1_ and its sister species D_8_, the two representative of subsection *Houzingenia* [30,39,42], both has four 45S loci on chromosomes D_1_07(D_8_07), D_1_09(D_8_09), D_1_03(D_8_03) and D_1_11(D_8_11) (Table 2 and Figure 4 [17]). The latter two 45S loci could be more unique to the two species rather than other species, which is in accordance with their clades showing different from other three clades in cladogram [24]. The rest six species, derived from the same clade but being divided into three subsection [24], have common 45S loci on chromosomes D_g_07, D_g_09 and D_g_05 supporting the same clade, however the variation of the last 45S locus distribution suggested two divisions among the six species as the following: On one hand, D_3-k_ and D_3-d_ of subsection *Integrifolia* Todaro and D_9_ of subsection *Erioxylum* all have four 45S rDNA on the chromosomes D_g_07, D_g_09, D_g_05 and D_g_12, suggesting that they might be closer than with other three species. And copy numbers of the 45S locus on chromosome D _3-d_09 is of much less than other nine species of D genome including D_3-k_ (Table 2 Figure 4 * [18]). On the other hand, the other three species, D_2-1_ of subsection *Caducibracteolata* Mauer as well as D_4_ and D_11_ of subsection *Erioxylum* , all have three 45S loci on the chromosomes D_g_07, D_g_09 and D_g_05 (Table 2 Figure 4 ** [17]), suggesting that they might be closer than their relationship previously reported [24].

#### Phylogenetic implication for African–Asian diploid species (A, B, E and F genome)

The A, B, E and F genomes belong to the same clade, which are different from other genomes, according to the phylogenesis history of *Gossypium* genus [24]. Species of these four genomes have same numbers of 45S and 5S loci (except E_4_) but varied significantly in synteny relationship. A and F genomes with the same rDNA pattern could associate with their proposed sister relationship [24]. And the 5S locus in F_1_ was found near the satellite outside the 45S rather than near centromere inside the 45S unlike other species. Philips [31] proposed that F_1_ should be removed from E genome and classified as F genome, as F_1_ could be a new cellular type according to cytology research. F_1_ with the synteny relationship of 5S and 45S is different from E genome with the nonsynteny relationship, providing more visual evidence of the further establishment of F genome. Besides, the 5S and 45S rDNA at telomere ends in F_1_ are close to each other, which is rare in *Gossypium* species though it has been reported in other plants [43–45], suggesting it may relate to the stabilization of centromeric fission products [45,46].

And, the similar distribution of 5S and 45S loci was observed in B_1_ and B_3_. It was not extensively accepted that B_3_ was classified into B genome at the early days. As far as phenotypic traits, B_3_ grows likewise A_1_ (with yellow crown, apetalous basis points and five-room capsule), other than any species of B genome (with ivory petal, large basis points of petals and three-room capsule). From the synteny relationship of 5S and 45S, B_3_ could be confirmed to be in B genome. And the nonsynteny relationship of 5S and 45S could be considered as the classification basis of B genome. So the rDNA identification for all species of B genome has the cytogenetic evidences for the classification of *Gossypium* genus. Notablely, the two 5S loci in E_4_ is a great discovery in *Gossypium* genus, suggesting special evolutional implication in species of E genome.

#### Phylogenetic implication for Australia diploid species (G genome)

The numbers of 45S rDNA were revealed three and four in G_3_ and G_1_, respectively, although the two G-genome species have similar morphological traits [47]. It is a certainty for G genome in terms of the taxonomy, which had been well studied [40,47]. Therefore, the origin might account for the difference in the rDNA numbers between two species. According to the cpDNA analysis, the chloroplast genome of G_1_ was similar with that of *G* *. sturtianum* , a morphologically distant C-genome species, suggesting that G_1_ could have a reticulate history with *G* *. sturtianum* .

In summary, the current study has clarified systematically the interrelationship of the rDNA distribution among 25 *Gossypium* species and one variant covering AD, A, B, D, E, F and G genomes. And the corresponding phylogenetic implications have been revealed for the evolution of *Gossypium* genus. Further study is needed to investigate the more precise rDNA patterns on meiotic pachytene chromosomes between with the development of cotton FISH techniques [48].
